# Introduction of cadmium chloride additive to improve the performance and stability of perovskite solar cells

**DOI:** 10.1039/d2ra03776a

**Published:** 2022-07-15

**Authors:** Mustafa K. A. Mohammed, Majid S. Jabir, Haider G. Abdulzahraa, Safa H. Mohammed, Waleed Khaild Al-Azzawi, Duha S. Ahmed, Sangeeta Singh, Anjan Kumar, S. Asaithambi, Masoud Shekargoftar

**Affiliations:** Radiology Techniques Department, Dijlah University College Al-Masafi Street Baghdad 00964 Iraq mustafa_kareem97@yahoo.com +964-7719047121; Applied Science Department, University of Technology Iraq; Department of Prosthodontic, Dijlah University College Al-Masafi Street Baghdad Iraq; Radiological Techniques Department, Al-Mustaqbal University College Babylon Iraq; Department of Medical Instruments Engineering Techniques, Al-Farahidi University Baghdad Iraq; Microelectronics Lab, National Institute of Technology Patna 800005 India; VLSI Research Lab, GLA University Mathura-281406 India; Department of Physics, Alagappa University Karaikudi 630 003 Tamil Nadu India; Laboratory for Biomaterials and Bioengineering (CRC-I), Department of Min-Met-Materials Engineering, Laval University Quebec City QC G1V0A6 Canada; University of Warith Al-Anbiyaa Karbala Iraq

## Abstract

With the increase in the importance of using green energy sources to meet the world's energy demands, attempts have been made to push perovskite solar cell technology toward industrialization all around the world. Improving the properties of perovskite materials as the heart of PSCs is one of the methods to fabricate favorable photovoltaic (PV) solar cells based on perovskites. Here, cadmium chloride (CdCl_2_) was used as an additive source for the perovskite precursor to improve its PV properties. Results indicated CdCl_2_ improves the perovskite growth and tailors its crystalline properties, suggesting boosted charge transport processes in the bulk and interfaces of the perovskite layer with electron–hole transport layers. Overall, by incorporation of 1.0% into the MAPbI_3_ layer, a maximum power conversion efficiency of 15.28% was recorded for perovskite-based solar cells, higher than the 12.17% for the control devices. The developed method not only improved the PV performance of devices but also boosted the stability behavior of solar cells due to the passivated domain boundaries and enhanced hydrophobicity in the CdCl_2_-based devices.

## Abstract 2

With the increase in the importance of using green energy sources to meet the world's energy demands, attempts have been made to push perovskite solar cell technology toward industrialization all around the world.

## Graphical abstract

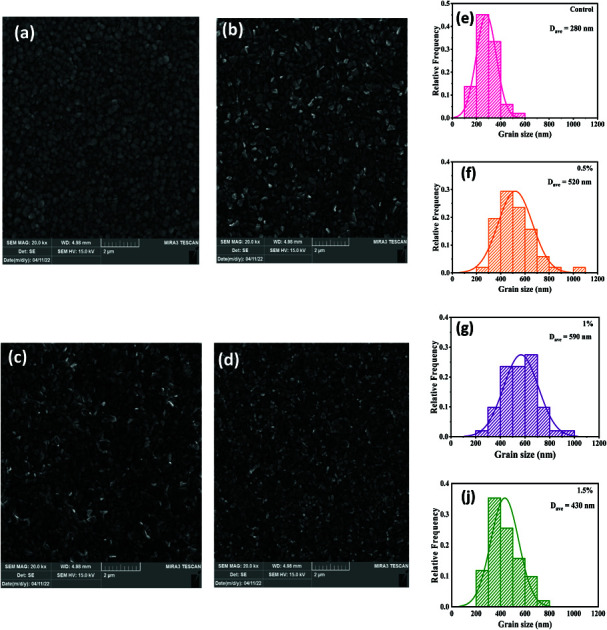

## 1. Introduction

Due to their exceptional photoelectric performance and easy fabrication processes, hybrid perovskite solar cells (HPSCs) have attracted considerable attention as a promising candidate for new-generation photovoltaic (PV) devices.^1–3^ This could be related to the high absorption coefficient, high charge transfer phenomenon, and adjustable bandgap along with high charge diffusion length, which are all important requirements.^4–6^ The efficiency of HPSCs has substantially increased from a low of 3.8% (in 2009)^7^ to a verified 25.2% (in 2021),^8^ which has encouraged researchers in the HPSC's future development. Typically, according to the direction of the photocurrent, the fundamental architecture of HPSCs can be categorized into conventional (n–i–p) and inverted (p–i–n) architectures.^9,10^ It's worth mentioning that the perovskite material is the heart of an HPSC device, and its overall quality determines how well it performs. The power conversion efficiency (PCE), hysteresis, and long-term stability of HPSCs are all influenced by the morphology, crystalline nature, and surface covering of the perovskite film.^11–13^ A high content of pinholes and grain boundaries (GBs) in the perovskite film, particularly, produces severe shorting and trap centers for non-radiative recombination, which is one of the primary sources of PCE degradation in HPSCs.^14,15^ Charge recombination aided by pinholes and GBs is also associated with abnormal hysteresis and instability concerns. As a result, enhancing the perovskite properties *via* passivating defects and optimizing perovskite quality has become a critical technique for decreasing recombination rates, removing unfavorable hysteresis, prolonging HPSC longevity, and increasing PCE.^16,17^

To date, several ways of controlling perovskite morphology have been explored, involving manipulating the deposition method, additive engineering, interfacial engineering, and compositional engineering.^18–22^ Additive engineering has been shown to be effective in improving the quality of the perovskite layer since it is a more simple and reproducible approach. Some agents, including copper chloride, *N*,1-diiodoformamidine, potassium hexafluorophosphate, formamidine acetate salt, *etc.*, can form an intermediate phase with perovskite precursors, which could regulate the dynamic process of crystal formation and thereby minimize GBs.^23–27^ The incorporation of cadmium (Cd^2+^) additive by Suneth *et al.* was a fundamental advance in controlling methylammonium lead iodide (MAPbI_3_) crystallinity using a two-step deposition method. With this treatment, the efficiency of MAPbI_3_-based HPSCs was significantly boosted from 7.1 to 13.8%.^28^ Yong *et al.* reported the addition of Cd^2+^ into organic–inorganic cation-based Cs_*x*_FA_1−*x*_PbI_3_ lattice to enhance the crystallinity and the charge carrier lifetime of perovskite film. An efficiency up to 20.59% was achieved for HPSC with a structure of ITO glass/SnO_2_/Cs_*x*_FA_1−*x*_PbI_3_/spiro-OMeTAD/Au.^29^ In another study, a suppression of atomic vacancies *via* strain relaxation of CsMAFA triple-cation perovskite film was reported by Makhsud *et al.* This suppression was achieved *via* incorporation of Cd isovalent small ions, which let to boost the stability of HPSCs in ambient air.^30^ Recently, Cd^2+^ introduced into all-inorganic perovskite for carbon-based HPSCs with a framework of FTO/SnO_2_/CsPbIBr_2_/carbon. They demonstrated that Cd ions improves the crystalline nature and morphological merits of perovskite film, resulting in better optical absorption and carrier transfer in perovskite with less recombination rates. Also, in compared to the HPSCs without Cd^2+^ doping (4.36%), the improved HPSCs attained the highest PCE of 6.79%.^31^ Yi *et al.* introduced ammonium benzenesulfonate as a passivator into the MAPbI_3_ solution to assist the film crystallization and mitigate trap density. A champion PCE of 20.6% is obtained for the modified HPSCs.^32^ Hua *et al.* reported the incorporation of *N*,1-diiodoformamidine modifier into FAMAPbI_3_ to improve the quality of perovskite with bigger grain size and fewer defects, hence improving the PCE from 19.07% to 21.22% as well as their light and thermal stabilities.^24^ Recently, a dual additive approach for enhancing the performance of HPSCs was reported by Yang *et al.*^33^ The highest PCE of 23.2% is yielded *via* a mixed modifier of MACl/CsCl with a 2 : 1 ratio, which is better than that for a single modifier of MACl or CsCl. Despite their high performance, the majority of HPSCs fabricated with organic hole transport layers (HTLs) such as spiro-OMeTAD have low stability and are excessively expensive, making them unsuitable for commercialization. Therefore, further work is needed to find a cheaper alternative to spiro-OMeTAD while simultaneously utilizing the additive engineering approach.

Herein, we take advantage of using an efficient additive of cadmium chloride (CdCl_2_) alongside with using a copper phthalocyanine (CuPc) semiconductor as a model HTL for regular (n–i–p) HPSCs. CuPc is known to possess high hole mobility, excellent interfacial bonding features, and superb stability while also being relatively inexpensive, which is why it has been used as a p-type semiconductor in different optoelectronic devices.^34–36^ We investigate the impact of employing low CdCl_2_ concentrations and high MAI contents during MAPbI_3_ development in the anti-solvent deposition procedure. In addition to better crystalline phase and increased grain size, steady-state photoluminescence (PL) tests revealed a significant reduction in charge recombination process. The best-performing CdCl_2_-based HPSCs using a CuPc HTL yielded a PCE of 15.28% with a *V*_OC_ of 1.0 V and a FF of 73.2%, recorded under reverse scanning. The modified devices demonstrated enhanced long-term stability as compared to unmodified devices fabricated with mesoscopic structures.

## 2. Experimental part

### 2.1 Materials

All solvents, titanium isopropoxide (TTIP, 97%), cadmium chloride (CdCl_2_, 99.99%), lithium bis(trifluoromethylsulfonyl)imide salt (LiTFSI, 98%), and copper phthalocyanine (CuPc, 99%) were provided from Sigma-Aldrich. Lead iodide (PbI_2_, 99.99%), methylammonium iodide (MAI, 99.5%), and 2,2′,7,7′-tetrakis(*N*,*N*-di-*p*-methoxyphenylamino)-9,9′-spirobifluorene (spiro-OMeTAD, 99.8%) were prepared from LumTec. TiO_2_ paste was prepared from Dyesol.

### 2.2 Solution preparation

922 mg of PbI_2_ were dissolved in 1.27 mL of *N*,*N*-dimethylformamide (DMF, 99.8%) and 0.142 mL dimethyl sulfoxide (DMSO, 99.8%). The resultant product was mixed at 80 °C for 45 min. After cooling down the PbI_2_ solution, 318 mg of MAI was inserted into it and stirred for 15 min to prepare MAPbI_3_. For incorporation of CdCl_2_ into the perovskite layer, different amounts of CdCl_2_ in molar ratios to MAI are added into the prepared perovskite solutions, followed by further stirring at RT for 60 min. 500 mg of TiO_2_ paste is dissolved in 3.0 g ethanol (EtOH, 99.8%) and is stirred at RT for 24 h to attain mesoporous TiO_2_ (mp-TiO_2_) solution. 700 mg of TTIP is added into 10 mL of 2-propanol (IPA, 99.9%) containing 70 μL of 2 M HCl, followed by stirring in an ice bath for 30 min to prepare compact TiO_2_ (c-TiO_2_) precursor. The spiro-OMeTAD precursor was prepared as reported in the literature of (ref. 37) by dissolving 36.2 mg of spiro-OMeTAD (99%) in 500 μL chlorobenzene (CB, 99.9%) doped with LiTFSI, and 4-*tert*-butylpyridine (98%).

### 2.3 HPSC fabrication

Transparent conductive F-doped SnO_2_ (FTO, Pilkington) glasses with a sheet resistance of 15 Ω sq^−1^ are ultrasonically cleaned with distilled water, soap, distilled water, acetone, and IPA successively for 10 min. 45 μL of the c-TiO_2_ solution is spread in each FTO piece and spin-coated at 4000 rpm, followed by annealing at 500 °C for 30 min to fabricate a c-TiO_2_ layer. 60 μL of mp-TiO_2_ precursor is spin-coated on each c-TiO_2_ layer at 4000 rpm for 30 s, followed by baking at 450 °C for 60 min. To fabricate perovskite layer, 60 μL of perovskite precursors with or without CdCl_2_ additive are spread on each substrate and spin coated at 1000 rpm for 8 s and then accelerate up to 6000 for 30 s. During the faster step, 200 μL of CB was pipetted on perovskite layer to assist crystal growth of perovskite layer. Perovskite layers were annealed for 30 min at 100 °C. The CuPc hole transport layer (HTL) with thickness of 30 nm was thermally evaporated on the pre-formed perovskite films in a vacuum of 10^−3^ Pa with deposition rate of 1 Å s^−1^. To fabricate spiro-OMeTAD HTL, its precursor was spin-coated at 5000 rpm for 30 s on top the perovskites. Finally, a 100 nm gold thin-layer was thermally deposited the HTLs.

### 2.4 Characterizations

A Mira3, TESCAN field emission scanning electron microscopy (FESEM) was used to monitor the effect of the CdCl_2_ additive on the micro-morphology of perovskite layers. A Bruker, D8 Advance X-ray diffractometer was used to collect XRD pattern of perovskite films. A LAMBDA 1050 spectrophotometer was used to study the light-harvesting ability of perovskite layers. A HORIBA Fluorolog-III spectrometer with an exciting wavelength at 405 nm was used to record steady-state photoluminescence (PL) responses of perovskite layers. Photovoltaic performance of MAPbI_3_-based solar cells with an active area of 2 mm × 3 mm was characterized using a Keithley Model 2401 under a calibrated AM 1.5 light simulator to record current density–voltage curves. A calibrated Newport IPCE equipment was employed to record incident photon-to-electron conversion efficiency (IPCE) of solar cells.

## 3. Results and discussion

**Fig. 1:**
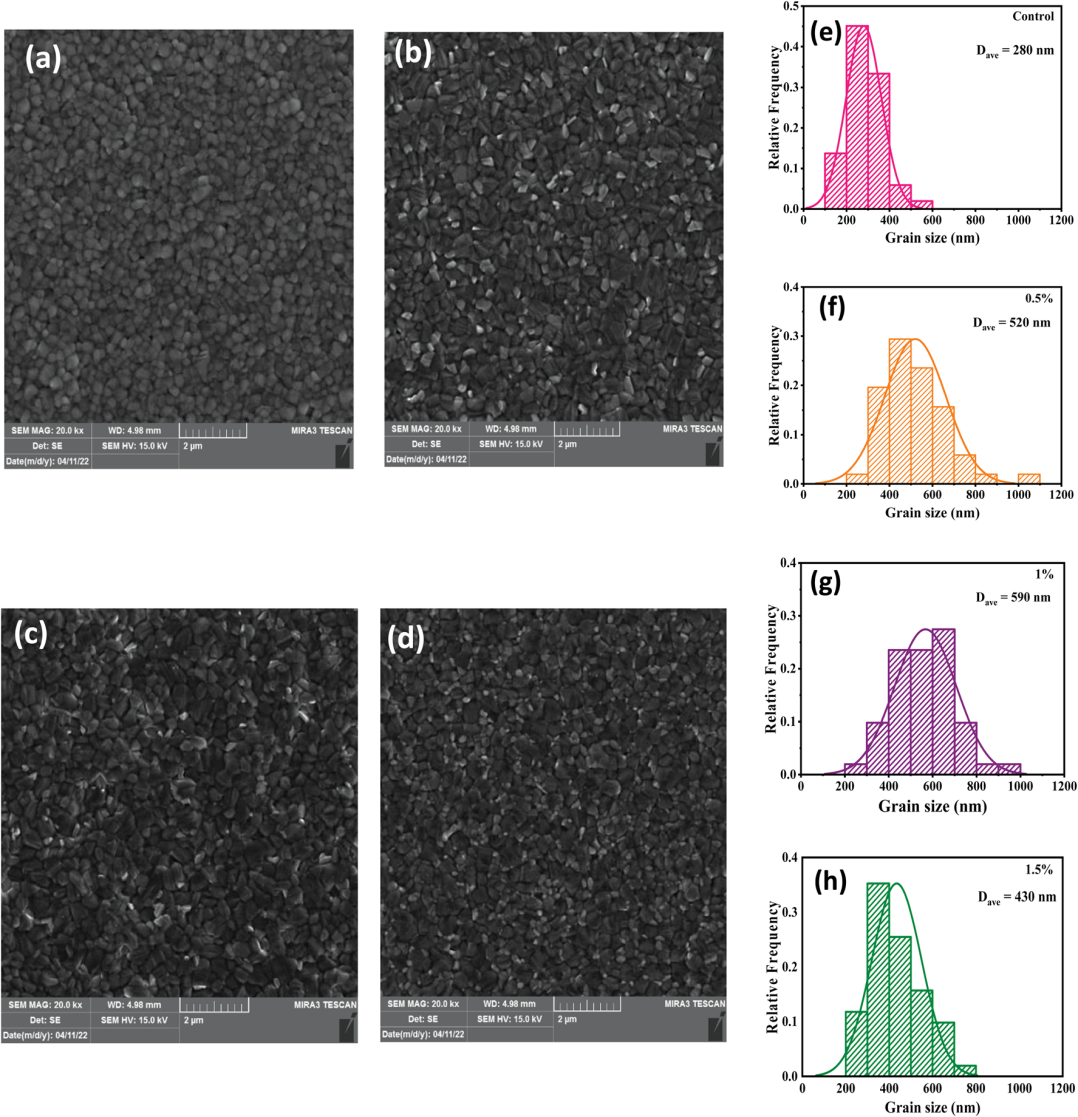
The top-view FESEM visualization of perovskite layers with (a) 0%, (b) 0.5%, (c) 1%, and (d) 1.5% of CdCl_2_ in perovskite precursors. Substrate for all layers was FTO/c-TiO_2_/mp-TiO_2_. (e–h) Particle size distributions of perovskite films evaluated by ImageJ software.

Fig. 1 presents the FESEM micrographs of the perovskites prepared with and without the CdCl_2_ additives in the MAI precursors that were utilized to complete the MAPbI_3_ conversion. The influence of the CdCl_2_ incorporation is clear in that good uniformity and large grains are observed in the CdCl_2_-added perovskites immediately after heat treatment (Fig. 1b and c), while film deposited without solvent additive shows low uniformity and small grains with obvious pinholes at the grain edges (Fig. 1a). As illustrated in Fig. 1e–h, the addition of small amounts of CdCl_2_ enlarged the average grain size fivefold, from 280 nm to 590 nm. This is due to the inclusion of CdCl_2_ into the MAI solution, which produces Cl ions, resulting in the formation of an intermediate phase that acts as a seed and enhances the development of bigger MAPbI_3_ crystals.^38^ With a further increase of CdCl_2_ amount to 1.5%, the surface of the perovskite layer was identical to the 1% CdCl_2_-added film but revealed small particles with obvious grain boundaries (GBs). From the FESEM results, we can argue that the grain size of the CdCl_2_-modified films was not greatly enlarged. Nevertheless, smaller grains have combined into bigger ones, the gaps between grains have been passivated, and the film has become denser and more compact. This could help improve HPSC performance by reducing nonradiative charge recombination.^39^

**Fig. 2:**
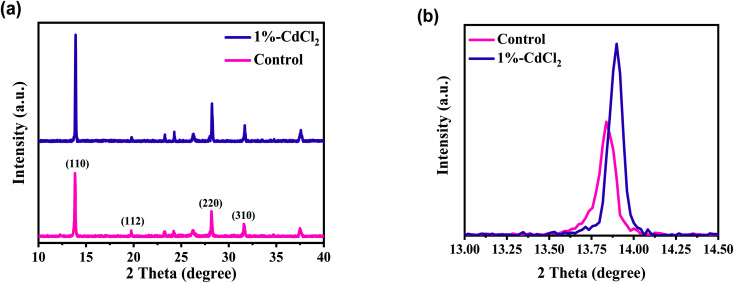
(a) XRD patterns of perovskite layers without (control) and with 1% of CdCl_2_ deposited on FTO/c-TiO_2_/mp-TiO_2_ substrate. (b) Zoomed XRD patterns around 2*θ* = 13.9°.

The performance of HPSCs is directly dependent on the crystal phase and purity of the MAPbI_3_ layers.^40^Fig. 2a demonstrates the X-ray patterns of MAPbI_3_ perovskites without and with 1% CdCl_2_ inclusion into the MAI solution. As demonstrated, multiple main XRD peaks at 13.82°, 20.10°, 28.18°, and 31.59° were indexed as (110), (112), (220), and (310) planes of the tetragonal MAPbI_3_ phase, respectively.^21^ The sharp and intensive peaks in the XRD plots of the MAPbI_3_ perovskites implied that the films were well crystallized. Furthermore, the intensities of the prominent peaks all rose when the CdCl_2_ solvent was incorporated, implying that a superior crystalline nature and larger crystallite size for perovskite film were developed as compared to a film prepared without CdCl_2_.^41^ In MAPbI_3_ samples prepared without and with CdCl_2_, the full width at half maximum intensity of the (110) peak was 0.108 and 0.097, respectively. According to the Scherrer equation,^42^ the average crystallite sizes of the corresponding samples were 73.46 and 81.21 nm, respectively. These findings suggest that the moderate amount of CdCl_2_ in perovskite could increase both crystallinity and grain size. This result is also in line with the findings of the FESEM images.

The low XRD signal at 12.4° is indexed as the cubic PbI_2_, because of the slight degradation of the MAPbI_3_ to PbI_2_ and/or incomplete crystallization of perovskite.^23^ The presence of PbI_2_ in the perovskite would be detrimental to photo-induced carrier transfer, lowering film stability and increasing the hysteresis behavior of HPSCs. Interestingly, it was apparently seen that the typical peak of PbI_2_ was completely removed with CdCl_2_ treatment. Fig. 2b shows the XRD patterns of perovskite samples zoomed to the major (110) peaks. The (110) peak was altered to higher values of 2*θ* with the addition of CdCl_2_. This is most likely owing to a variation in the lattice parameters caused by the replacement of smaller Cd^2+^ species for Pb^2+^ sites,^28^ therefore leading to the shrinkage of the MAPbI_3_ lattice. The reduced lead amounts in perovskite could be beneficial for increasing the tolerance factor, improves the stability of the MAPbI_3_ structure.^31^

**Fig. 3:**
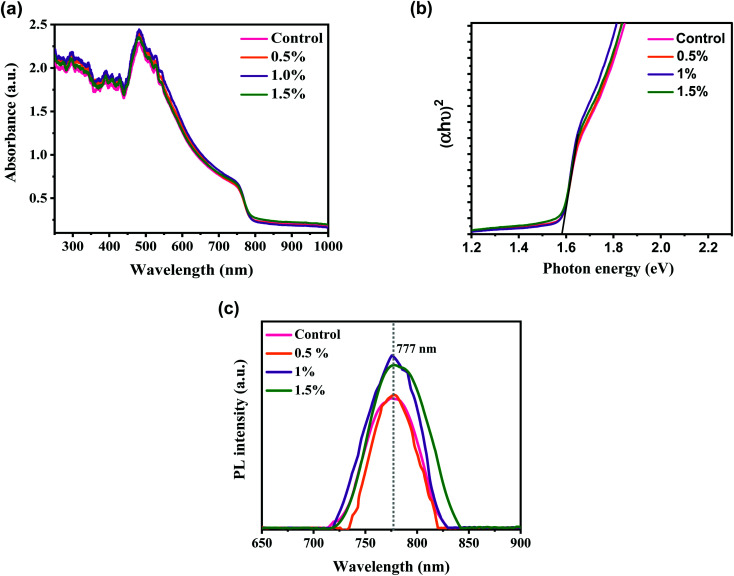
(a) UV-vis absorption spectra, (b) Tauc plots, and (c) photoluminescence spectra of control and modified perovskite layers with different amounts of CdCl_2_, deposited on glass substrates.

We used UV-vis absorption and PL spectroscopy to explore the influence of the CdCl_2_ solvent on light absorption and the mechanism of photoexcited carriers transport in perovskite film deposited under the same conditions unless the variation in CdCl_2_ concentrations in MAI solution. The UV-vis spectra of MAPbI_3_ perovskite layers with various concentrations of CdCl_2_ additive are shown in Fig. 3a. The absorbance plots measured for MAPbI_3_ layers with and without CdCl_2_ treatments exhibited remarkably identical absorption profiles (edge-absorption position at 777 nm) thanks to the unique energy bandgap (*E*_g_) absorption of MAPbI_3_ as a sunlight harvester.^43^ As we can see, all samples fabricated by the spin-coating clearly show strong absorbance over the full light absorption below 700 nm. When the concentration of CdCl_2_ solution is raised to 1%, the absorption performance of the associated MAPbI_3_ layer slightly increases within the visible range. This could be due to the increased grain size and improved crystallinity, while the film thickness remains similar with the insertion of CdCl_2_.^44^ However, when more CdCl_2_ (1%) was introduced, the perovskite's light absorption capacity dropped because the presence of unwanted defects and GBs disrupted the film's compact structure. Fig. 3b describes the Tauc plots for all samples prepared with and without CdCl_2_ additives. The value of *E*_g_ can be evaluated by extrapolating the variation between photo energy (*hν*) and (*αhν*)^2^.^45^ The values of *E*_g_ were found to be 1.59 eV, which is similar to the values reported in the literature.^13,40^

Fig. 3c shows the steady-state PL measurements of perovskite films spin-coated on glass substrates without HTL and ETL films in order to reduce charge extraction at the interfaces and enable the inherent features of the MAPbI_3_ film to govern the recombination dynamics. All the steady-state PL plots of perovskites reveal a typical and strong luminescence peak at locations identical to the absorbance edges detected in UV-vis measurements. The increased PL intensity and reduced linewidth of CdCl_2_-treated films in comparison to the control layer indicate that the incorporation of solvent additive can significantly inhibit trap-mediated, non-radiative recombination in those perovskites.^46^ This could be due to the CdCl_2_ additive treatments improving the crystallinity of the developed perovskites. The enhanced crystalline structure decreased defects in the entire MAPbI_3_, resulting in fewer recombination taps in the film. This inhibited the non-radiation recombination pathway, making it more suitable for optoelectronics manufacturing. Conversely, as the CdCl_2_ concentration was increased to 1.5%, the PL intensity of the perovskite decreased, indicating an increase in the density of microstructural defects.

**Fig. 4:**
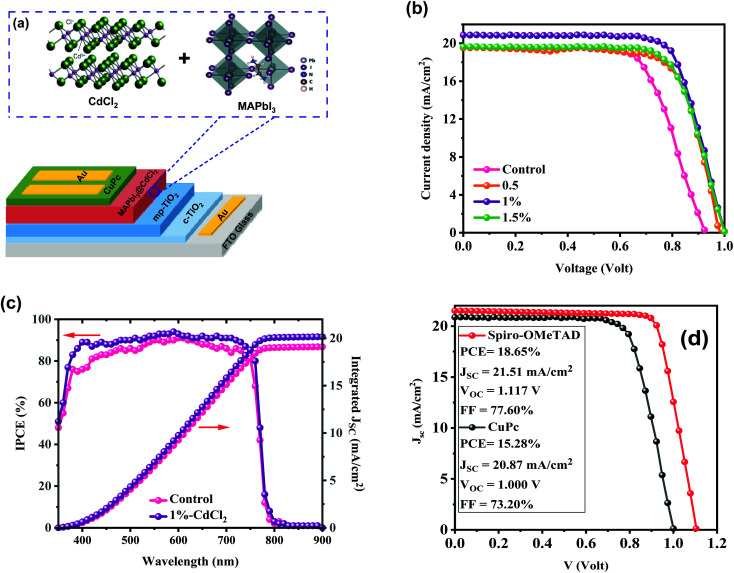
(a) Schematic diagram of the device architecture of CdCl_2_-assisted deposition process for MAPbI_3_ film. (b) *J*–*V* curves of the best-performing HPSCs with different amounts of CdCl_2_ additive. (c) Recorded IPCE responses of the control and modified device with 1% of CdCl_2_ and their integrated *J*_SC_ from the IPCE. (d) *J*–*V* curves of perovskite solar cells with copper phthalocyanine (CuPc) and 2,2′,7,7′-tetrakis(*N*,*N*-di-*p*-methoxyphenylamino)-9,9′-spirobifluorene (spiro-OMeTAD) as hole transport layers.

We fabricated regular (n–i–p) HPSC devices using the architecture of glass/FTO/c-TiO_2_/mp-TiO_2_/MAPbI_3_/CuPc/Au, as illustrated in Fig. 4a. The ETL films are fabricated based on our previously prepared mp-TiO_2_*via* the spin-coating method, while HTLs utilize a p-type inorganic film named CuPc. The mixed solutions of MAI : PbI_2_ were used to form perovskite film with an anti-solvent assistance approach. An additive engineering method was employed in our perovskite films to improve the HPSC performance. The CdCl_2_ additive was selected as a modifier to assist perovskite crystallization during the deposition process. We have investigated the PV parameters of our designed HPSCs with reverse *J*–*V* sweep being performed under the AM 1.5 sunlight simulator. Fig. 4b demonstrates the best-performing *J*–*V* plots of the HPSCs fabricated with and without additive treatments, and the summarized cell parameters are displayed in Table 1. The performance of HPSCs with and without CdCl_2_ additives varies significantly. Without CdCl_2_, the HPSCs show low PV performances with a *J*_SC_ of 19.78 mA cm^−2^, *V*_OC_ of 0.962 V, FF of 65.70% and, a champion PCE of 12.17%. The HPSC has an enhanced PCE of 15.28% with a high *J*_SC_ of 20.87 mA cm^−2^, a *V*_OC_ of 1.00 V, and a good FF of 73% after adding 1% CdCl_2_. When the amount of CdCl_2_ is increased to 1.5%, the PCE of the HPSC has a notable reduction to 14.10% with reduced *J*_SC_ and practically unchanged *V*_OC_. As previously mentioned, an adequate dose of CdCl_2_ modifier can assist in removing PbI_2_ residual without forming a non-perovskite phase, resulting in a compact high-quality layer with large crystals, which can greatly reduce interior recombination and enhance HPSC performance. The integrated *J*_SC_ values of the unmodified and 1% CdCl_2_-based HPSCs are 19.08 mA cm^−2^ and 20.14 mA cm^−2^, respectively, as shown in Fig. 4c, which match with the *J*_SC_ values determined from the detailed *J*–*V* scan characteristics. The IPCE measurements confirm the accuracy of our HPSC efficiency characterizations.

**Table 1:** Photovoltaic properties of HPSCs with various concentrations of CdCl_2_ additives in MAI precursor

CdCl_2_ amount		*V*_OC_ a (V)	*J*_SC_ b (mA cm^−2^)	FF^c^ (%)	PCE (%)
Control	Average	0.946 ± 0.018	19.16 ± 0.48	63.26 ± 2.75	11.44 ± 0.63
	Best	0.962	19.78	65.70	12.17
0.5%	Average	0.964 ± 0.019	19.48 ± 0.55	69.15 ± 1.97	12.98 ± 0.52
	Best	0.987	19.54	71.70	13.82
1.0%	Average	0.994 ± 0.007	20.33 ± 0.41	72.29 ± 1.34	14.61 ± 0.43
	Best	1.000	20.87	73.20	15.28
1.5%	Average	0.985 ± 0.013	19.23 ± 0.53	69.68 ± 1.29	13.19 ± 0.50
	Best	1.000	19.64	71.80	14.10

a *V*_OC_ is open-circuit voltage.

b *J*_SC_ is short-circuit current density.

c FF is fill factor.

To compare the performance of CuPc-based PSCs with conventional spiro-OMeTAD PSCs, we fabricated PSCs with spiro-OMeTAD HTL. A champion PCE of 18.65% with a *J*_SC_ of 21.51 mA cm^−2^, *V*_OC_ of 1.117 mV, and FF of 77.60% was obtained (Fig. 4d). It notes that CuPc can be considered a low-cost HTL in perovskite-based solar cells. The CuPc HTL recorded a *J*_SC_ near to spiro-OMeTAD devices, with lower *V*_OC_ and FF than spiro-OMeTAD HTL one. Finding refers that recording higher PCE with CuPc HTL is reachable by more engineering at the interface of perovskite/CuPc, which will be targeted in our future experiments.

**Fig. 5:**
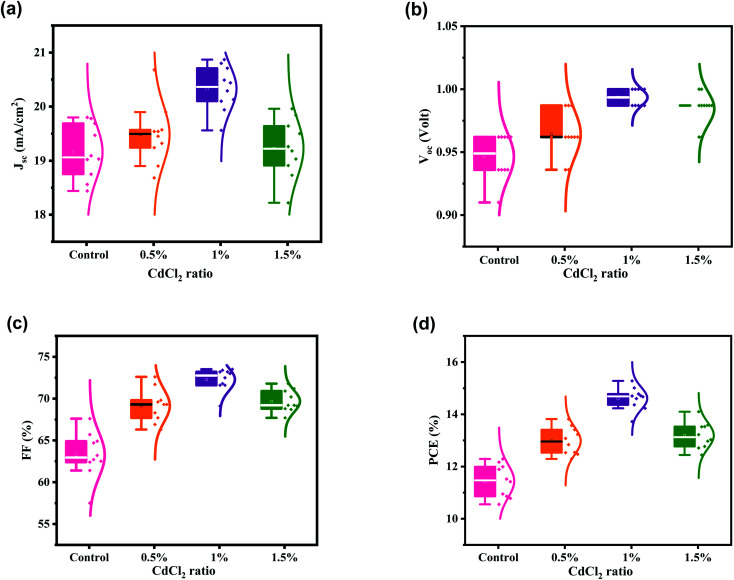
(a–d) Statistics of the PCE, *J*_SC_, *V*_OC_ and FF for HPSCs modified with different amounts of CdCl_2_ additive. PV parameters collected from 10 devices for each set.

Fig. 5 depicts the distribution of PV parameters depending on the concentration of MAI to CdCl_2_, where the ratio of MAI : CdCl_2_ is changed from 0% to 1.5%. As shown in Fig. 5a, adjusting the ratio has a notable effect on the mean *J*_SC_ of nearly 20 mA cm^−2^. *V*_OC_ improves slightly with increasing the CdCl_2_ concentration (Fig. 5b). The average *V*_OC_ value of 0.946 V for the control device is increased to 0.964, 0.994, and 0.985 V as the ratio varies from 0% to 1.5%. Mean FF is also enhanced as the concentration of CdCl_2_ increases, reaching 72.29% at 1% and then decreasing to 69.68% at 1.5% (Fig. 5c). As a consequence, PCE is boosted from 11.44% for the control device to 14.61% for the 1% CdCl_2_-modified device (Fig. 5d). Also, the statics show the narrow distribution of PV parameters of HPSCs based on the optimized 1% CdCl_2_, indicating excellent reproducibility of solar cells.

**Fig. 6:**
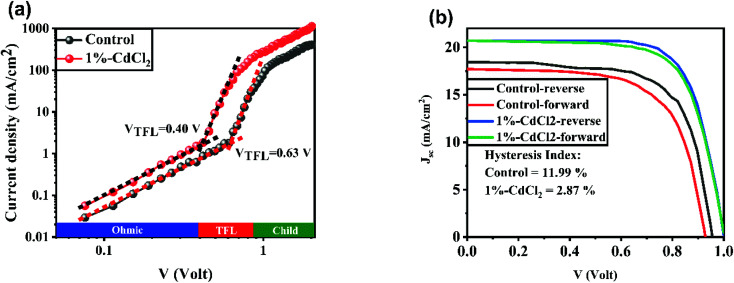
(a) *J*–*V* curves of electron-only of control and 1%-CdCl_2_ perovskite solar cells devices. (b) *J*–*V* curves of a perovskite solar cell device with or without of CdCl_2_ additive in reverse and forward scan directions.

To further study the role of CdCl_2_ material on the PV properties of space-charge-limited current (SCLC) measurements for electron-only devices with a structure of FTO/c-TiO_2_/mp-TiO_2_/MAPbI_3_ with or without CdCl_2_/PCBM/Au were investigated. The electron trap density (*N*_t_) of the perovskites layers were calculated as follows:^47^1
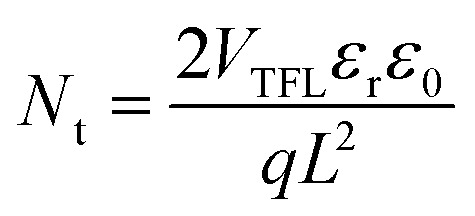
where *q* is the electric charge, *L* indicates the thickness of the perovskite film, *ε*_r_ is the relative dielectric constant of the MAPbI_3_ perovskite (*ε*_r_ = 32), *ε*_0_ refers to the free space permittivity, and *V*_TFL_ indicates for the trap-filled limit voltage. The *V*_TFL_ for the electron-only devices for the control and 1%-CdCl_2_ the 2D/3D perovskite layer were found to be 0.63 V and 0.40 V respectively. The hole trap density values for the control and 1%-CdCl_2_ PSCs are 13.19 × 10^15^ cm^−3^ and 8.38 × 10^15^ cm^−3^ respectively. It indicates that the CdCl_2_ additive facilitates the electron transfer process and reduces the charge recombination by suppressing the carrier traps within the bulk of perovskite layer. In addition, the hysteresis index (HI) of PSCs was recorded by measuring *J*–*V* curves in reverse and forward scan directions (Fig. 6b). HI was obtained using a formula of 
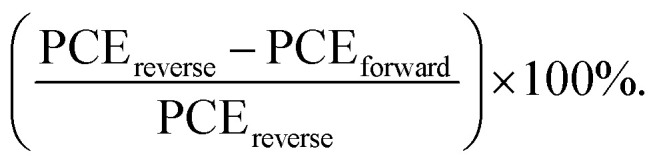
^40^ Compared with the control PSCs with a HI value of 11.99%, the 1% CdCl_2_-modified PSCs showed a HI of 2.87%. The weaken HI in the CdCl_2_-based PSCs can be attributed to the passivated domain boundaries in the modified perovskite layer. In addition, as SCLC results showed, CdCl_2_ incorporation into the perovskite layer accelerates electron transfer within the bulk of perovskite layer and suppresses HI in the PSCs.^48,49^

**Fig. 7:**
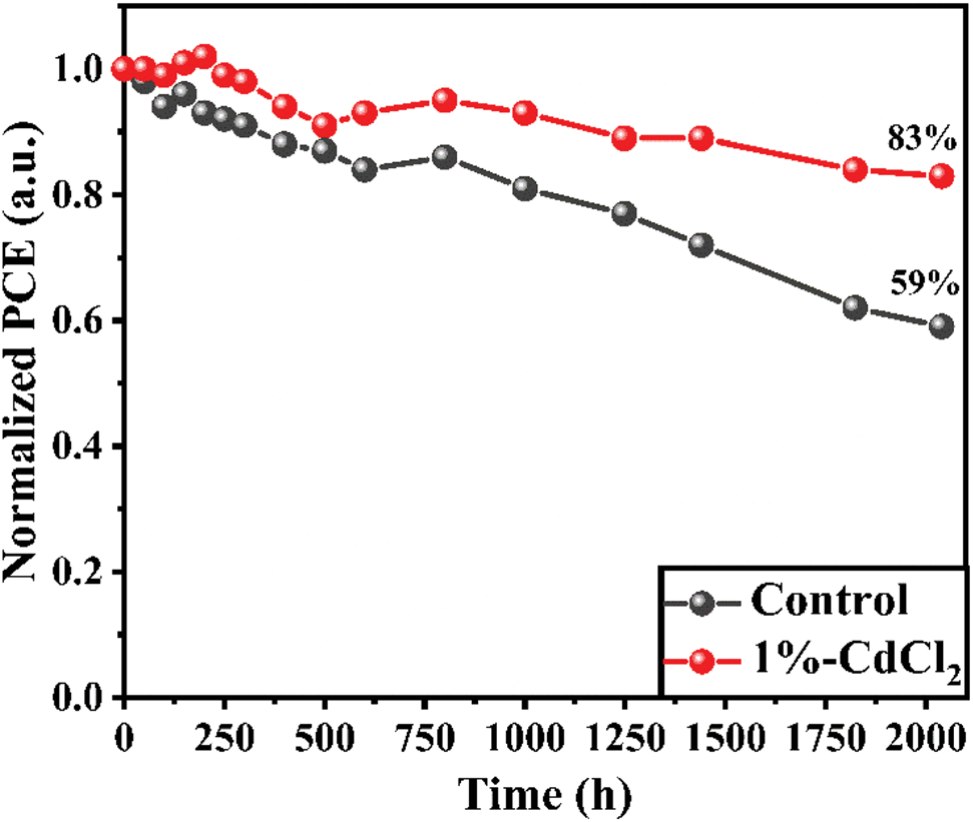
Stability test of the unsealed control perovskite solar cells stored in ambient conditions with a relative humidity (RH) of 25–40%.

One of the most critical performance factors for future commercial uses of HPSCs is long-term stability. Therefore, the HPSC longevity of related MAPbI_3_-based regular HPSCs (unmodified and with 1% CdCl_2_) was assessed by tracing the PCE progression under ambient conditions with a RH of 25–40% in the dark (see Fig. 7). After 2040 h of age, the PCEs of the HPSC with 1% CdCl_2_ additive demonstrate outstanding stability, maintaining 83% of the original value. In contrast, the performances of the unmodified HPSC dropped to less than 59% of their initial values after 2040 h, mainly attributable to perovskite degradation. Hence, the incorporation of CdCl_2_ in MAPbI_3_-based HPSCs, results in outstanding PV parameters and environmental stability.

## 4. Conclusion

In conclusion, we have proven a simple method based on additive engineering to improve the PCE and stability of MAPbI_3_-based HPSCs by adding a certain concentration of CdCl_2_ additive. The impacts of varying concentrations of CdCl_2_ additives on perovskite layer characteristics and PV properties were studied. High crystallinity, large grain size, improved optical absorption ability, and fewer pinholes and GBs were obtained for perovskite film fabricated with the optimized CdCl_2_ concentration, resulting in excellent photovoltaic performance. The synergistic influence of crystal growth control and pinhole passivation by CdCl_2_ effectively decreases the charge carrier recombination. With the incorporation of 1% CdCl_2_ into the MAI solution, the HPSC yielded a champion PCE of over 15.28% under the reverse scan conditions. In comparison with unmodified HPSC, the HPSC based on 1% CdCl_2_ also shows excellent ambient stability, which maintained 83% of its initial PCE after being stored for 2040 h without encapsulation. Our findings indicate that CdCl_2_ is a good additive for making inexpensive HPSCs with excellent stability.

## Conflicts of interest

The authors declare no conflict of interest.

## Supplementary Material
